# Deformable multi-level feature network applied to nucleus segmentation

**DOI:** 10.3389/fmicb.2024.1519871

**Published:** 2024-12-09

**Authors:** Shulei Chang, Tingting Yang, Bowen Yin, Jiayi Zhang, Liang Ma, Yanhui Ding, Xiaodan Sui

**Affiliations:** School of Information Science and Engineering, Shandong Normal University, Jinan, China

**Keywords:** nucleus segmentation, pathology images, deep learning, convolutional neural network, deformable multi-level feature network

## Abstract

**Introduction:**

The nucleus plays a crucial role in medical diagnosis, and accurate nucleus segmentation is essential for disease assessment. However, existing methods have limitations in handling the diversity of nuclei and differences in staining conditions, restricting their practical application.

**Methods:**

A novel deformable multi-level feature network (DMFNet) is proposed for nucleus segmentation. This network is based on convolutional neural network and divides feature processing and mask generation into two levels. At the feature level, deformable convolution is used to enhance feature extraction ability, and multi-scale features are integrated through a balanced feature pyramid. At the mask level, a one-stage framework is adopted to directly perform instance segmentation based on location.

**Results:**

Experimental results on the MoNuSeg 2018 dataset show that the mean average precision (mAP) and mean average recall (mAR) of DMFNet reach 37.8% and 47.4% respectively, outperforming many current advanced methods. Ablation experiments verified the effectiveness of each module of the network.

**Discussion:**

DMFNet provides an effective solution for nucleus segmentation and has important application value in medical image analysis.

## 1 Introduction

The nucleus plays an important role in the examination of hematoxylin and eosin stained tissue sections.Nuclear morphometric features and appearance, including the color of the surrounding cytoplasm, also help in identifying various types of cells, such as epithelial (glandular), stromal, or inflammatory cells, which in turn, provide an understanding of the glandular structure and disease presentation at low power (Kumar et al., 2017). In disease diagnosis, nuclear characteristics are key indicators. For example, abnormal morphological and structural changes in cancer cell nuclei, such as nuclear enlargement and nuclear-cytoplasmic ratio imbalance, can assist doctors in determining the type and stage of cancer. Moreover, nuclear segmentation can also contribute to pathological research by enabling the understanding of cellular level changes during the development of diseases. Therefore, accurate nucleus segmentation is critical in the field of medicine.

Owing to the importance of nuclear information in medicine, numerous researchers have proposed pathology image segmentation methods (Xinpeng et al., 2020; Liu et al., 2021), including level sets (Peifang et al., 2016), graphbased segmentation (Fuyong and Lin, 2016), mathematical morphologies (Wang et al., 2016), and pixel classification (Liu et al., 2019). However, such methods fail to generalize across a wide spectrum of tissue morphologies due to inter- and intra-nuclear color variations in crowded and chromatin sse nuclei (Kumar et al., 2017). Traditional methods face numerous limitations, and under these circumstances, machine learning techniques have gradually become a new hope for solving the nuclear segmentation problem due to their unique advantages. Techniques based on machine learning can provide better results for challenging cases of nucleus segmentation because they can be trained to recognize nucleus shapes and color variations (Yiming et al., 2018; Xieli et al., 2019).

However, automatic nucleus segmentation continues to be very challenging owing to variations in the nuclei, ambiguous borders, and differences in staining conditions. Nucleus segmentation tasks are challenging in three respects. Firstly, the shapes of the nuclei in the pathology images vary in shapes; however, the convolution kernel of the convolutional neural network (CNN) modules is a fixed geometric structure. In other words, the CNN modules do not possess the internal mechanism to handle nuclei of different shapes. Secondly, deep high-level features in the backbones have more semantic meanings, while shallow low-level features are more content descriptive (Zeiler and Rob, 2014). For example, high-level information can provide many semantic details, like staining conditions. Low-level information can provide content, such as the location of the nucleus. Thirdly, most instance segmentation methods based on CNN comprise two stages, which are complex and have room for improvement in accuracy.

Over the past dozen years, deep learning has emerged as a prominent category of machine learning algorithms, including natural language processing, computer vision, and more. One of the most representative models in deep learning models is CNN. In computer vision, different locations on images may correspond to objects with different scales or deformation (Jifeng et al., 2017); for example, fully convolutional networks (FCNs) (Jonathan et al., 2015) provide semantic segmentation with the ability of adaptive determination of scales or receptive field sizes for visual recognition tasks with fine localization; however, their performance warrants further improvements. Feature integration has led to the development of instance segmentation. FPN (Tsung-Yi et al., 2017) and PANet (Shu et al., 2018) integrate features via lateral connections to achieve excellent performance; however, they cannot merge shallow and deep information with each other. AdaptIS (Konstantin et al., 2019) predicts point proposals for classagnostic instance segmentation, and then generates a mask for the object located at this point. PolarMask (Xie et al., 2020) uses instance center classification and dense distance regression in a polar coordinate system to predict the contour of instances. These methods may be considered as a semidirect adigm. They are anchor-free and make the CNN simple; however, all of them require additional complex processing methods. ooTensorMask (Xinlei et al., 2019) operates in a dense sliding window and segments objects in fixed local patches, limited by patch scale. SOLO aims to segment instance masks directly, under the supervision of full instance mask annotations rather than in-box masks or additional pixel pairwise relations (Xinlong et al., 2020).

Recently, the most common instance segmentation method is the two-stage method. It has two approaches. The first one is “detect then segment”. It first detects the target to create bounding boxes and then divides the mask in each box. The second one is “label then cluster”. First, each pixel is predicted, and then the pixels of the same instance are grouped together. This approach is usually not as effective as the first approach. A typical example of a two-stage approach is Mask R-CNN, which uses a region proposal network (RPN) (Shaoqing et al., 2017) to obtain and classify candidate regions, which are then segmented using an FCN (Jonathan et al., 2015) model. Two stage methods achieve both step-wise and indirect object localization and mask generation, which either rely heavily on bounding box detection or clustering. On the contrary, one-stage instance segmentation methods can simultaneously achieve object localization and mask generation. SOLO (Xinlong et al., 2020) is one of the representative methods of one-stage instance segmentation methods, which takes an image as input and directly outputs instance masks and corresponding class probabilities, using a fully convolutional, frameless and groupless adigm.

Faisal et al. (2020) enhanced structured prediction capabilities for nucleus segmentation through conditional generative adversarial networks trained with synthetic and real data. Peter et al. (2019) formulated the nuclear segmentation task as the regression of intra-nuclei map distance to solve the joint segmentation of close nuclei. Similar to the nucleus segmentation task, Hao et al. (2016) proposed a deep contouraware network integrating multiple layers of contextual features to accurately segment glands from pathological images. Carsen et al. (2021) proposed a segmentation method called Cellpose that can accurately segment cells from various image types, with exciting results. For better generating bounding box proposals, Jingru et al. (2019) proposed a keypoint-based detector combined with cell instance segmentation. Oskar et al. (2019) segmented nuclei based on Mask R-CNN and used bounding boxes to detect nuclei instances. However, the shape of the nucleus tends to be oval, which presents an occlusal problem. This means that each bounding box may contain pixels representing two or more instances, which suggests that the bounding box may end up being suboptimal for kernel segmentation (Shengcong et al., 2020). Ortiz et al. (2020) proposed an instance segmentation method based on a recurrent residual network, which offers the advantages of improved segmentation accuracy and enhanced feature propagation stability. However, the method has some drawbacks, including high computational cost and training time, limited flexibility when handling complex scenarios, and sensitivity to the quality of input data. In recent years, methods based on Transformer have gradually emerged. Chen et al. (2021) combined the Transformer encoder with the U-Net architecture for medical image segmentation, which is especially suitable for medical image segmentation tasks. This method can effectively capture long-distance global dependencies and improve the segmentation accuracy. However, the computational resource consumption of Transformer is relatively large, resulting in long training time and high memory requirements. Cao et al. (2022) enhanced the segmentation performance of medical images by introducing Swin Transformer and utilizing its multi-scale characteristics, especially performing prominently in medical image segmentation tasks. However, when dealing with very large or complex images, the local windowing method of Swin Transformer may limit the ability to extract global information. He et al. (2023) enhanced the model's ability by introducing convolution operations in SwinUNETR. However, the training process may be relatively complex, involving a variety of techniques and adjustments, which may increase the implementation difficulty in practical applications.

In this study, we introduce a novel method called the deformable multi-level feature network (DMFNet) for nucleus segmentation. The DMFNet is based on a CNN using images of H&E stained tissue specimens. The DMFNet employs two levels to process the features and masks separately. To address the three challenges mentioned earlier, the DMFNet effectively combines deformable convolutional networks (DCNs) (Jifeng et al., 2017), balanced feature pyramid (BFP) (Jiangmiao et al., 2019), and segmenting objects by locations (SOLO) (Xinlong et al., 2020). Even though each of these components has been used in the past, we demonstrate that their combination in nuclei segmentation is superior to the existing standard methods. Thus, the main contributions of this study are as follows:

First, we use a novel module to replace the feature extraction module of a conventional CNN for dense spatial transformations; this can increase the transformation modeling capability. In the new module, the convolution kernel can be in various forms of deformation for free sampling.

Second, we integrate multi-level features, which are rescaled, integrated, refined, and strengthened to obtain balanced semantic features and refine the same. Finally, we use a one-stage network to directly distinguish instances by the center locations and object sizes instead of masks in boxes or pixel-pairwise relations. Nuclei segmentation by location renders the segment framework simple, and flexible. The DMFNet achieved the best performance on the MoNuSeg 2018 dataset, with its mAP and mAR approaching 37.8% and 47.4%, respectively.

## 2 Materials and methods

**Figure 1:**
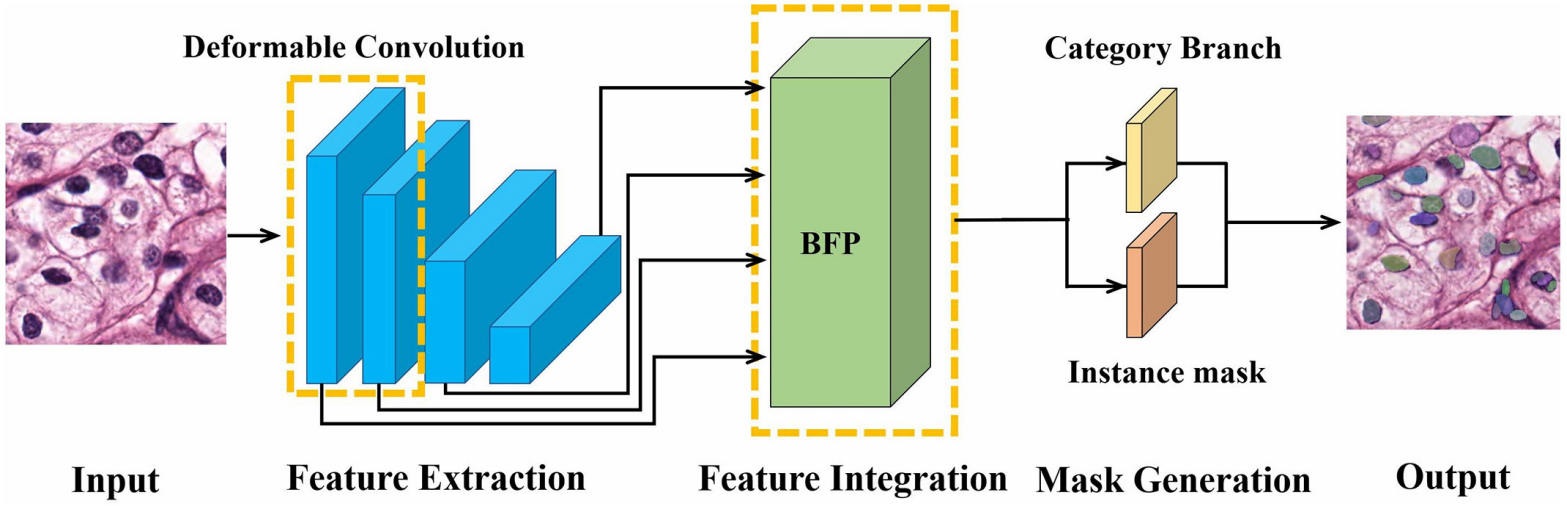
Illustration of our proposed DMFNet architecture.

SOLO is a one-stage algorithm, and its backbone comprises a residual network (ResNet) (Kaiming et al., 2016) and FPN (Tsung-Yi et al., 2017). To mitigate the complex structure in the two-stage methods, our DMFNet is based on SOLO for nucleus segmentation. The proposed model consists of a feature level and a mask level, as shown in Figure 1. Specifically, the input image is first extracted with features in ResNet, which incorporates a deformable convolution (Jifeng et al., 2017). Then, the features are integrated into a balanced feature pyramid to obtain enhanced multi-level semantic features. Finally, a mask generation network predicts categories and generates instance masks simultaneously, thus achieving an effective instance segmentation under a one-stage network.

### 2.1 Feature extraction

At the feature level, a deformable residual network is trained for an efficient feature extraction. The standard convolution consists of the following two steps: (1) use a regular grid R for sampling on the input feature map x; and (2) perform a weighting operation. For example,


(1) $$\begin{array}{l} R=\left(-1,-1\right),\left(-1,0\right),...,\left(0,1\right),\left(1,1\right) \end{array}$$

where *R* defines the size and dilation. Here, it defines a 3 × 3 kernel with a dilation of 1. Each position *p*_0_ on the output feature map *y*, is calculated using the following formula:


(2) $$\begin{array}{l} y\left(p_{0}\right)=\underset{p_{n}\in \mathbb{R}}{\sum }w\left(p_{n}\right)\cdot x\left(p_{0}+p_{n}\right) \end{array}$$

where *w* is the weight of the sampled values, and *p*_*n*_ is an enumeration of the locations listed in *R*. In this network, the regular grid R is expanded by adding offsets, where *N* = |*R*|. The same position *p*_0_ becomes:


(3) $$\begin{array}{l} y\left(p_{0}\right)=\underset{n\in \mathbb{R}}{\sum }w\left(p_{n}\right)\cdot x\left(p_{0}+p_{n}+\delta p_{n}\right) \end{array}$$

Now, the sampling location has become an irregular location. As the offset δ*p*_*n*_ is usually a decimal number, Equation (3) is implemented via a bilinear interpolation, shown in Equation (4) below. Here, *p* defines an arbitrary location and q is an enumeration of all the integral spatial locations listed in feature map.


(4) $$\begin{array}{l} x\left(p\right)=\underset{q}{\sum }G\left(p,q\right)\cdot x\left(q\right) \end{array}$$

where *G*(., .) denotes a bilinear interpolation kernel. It is divided into two one-dimensional kernels as follows:


(5) $$\begin{array}{l} G\left(p,q\right)=g\left(q_{x},p_{x}\right)\cdot g\left(q_{y},p_{y}\right) \end{array}$$

where *g*(*a, b*) = max(0, 1−|*a*−*b*|).

**Figure 2:**
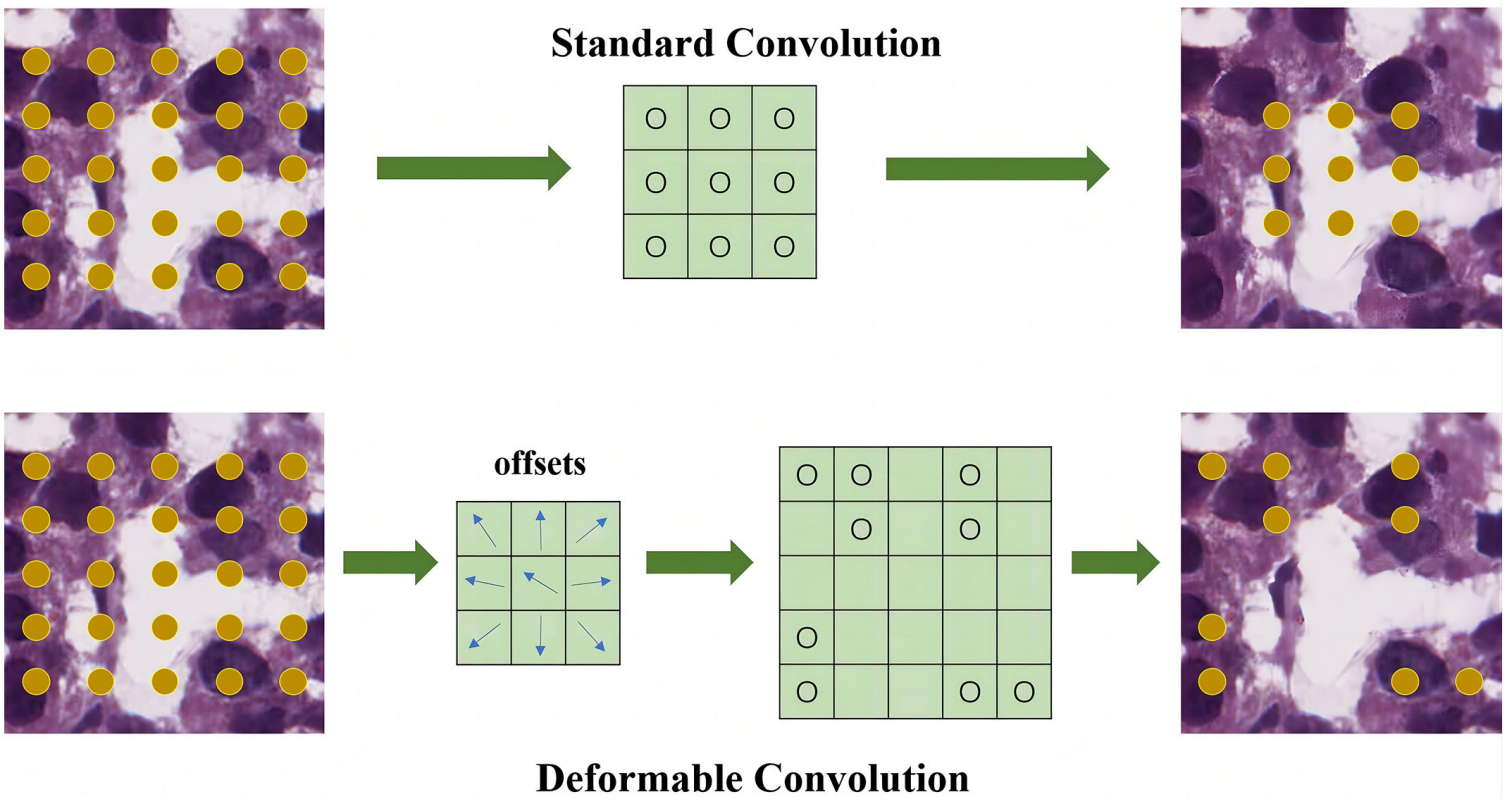
Feature extraction module for feature level. The upper part is the standard convolution, and the lower part is the deformable convolution.

As illustrated in Figure 2, the offsets are obtained by applying a convolutional layer over the same input feature map. To learn the offsets, the gradients are back propagated using Equations 4, 5. The deformable network is integrated with the state-ofthe-art architecture ResNet to enhance the capability of the DMFNet for modeling the transformations. This possesses excellent feature extraction capability for nuclei of various shapes.

### 2.2 Feature integration

Feature integration occupies a crucial position in the field of deep learning. It focuses on merging and summarizing the feature information obtained from different network layers, diverse functional modules, or various feature extraction methods, ultimately creating a more comprehensive expression that accurately depicts the target features. This technique plays a significant role in enhancing the model's ability to understand and process complex data. The Balanced Feature Pyramid is an innovative structure specifically designed to optimize the feature integration process. In the ongoing evolution of deep learning models, while traditional feature pyramid networks are capable of capturing multi-scale features, they often face the challenge of imbalanced information distribution when merging features from different levels. For example, in tasks such as cell nucleus segmentation, this imbalance can lead to inaccurate and incomplete descriptions of nuclear features. In response to this challenge, the Balanced Feature Pyramid was developed, with its core mission being to address this issue. Through a series of unique designs and operations, it makes the feature integration process more efficient and precise, thus providing a higher-quality feature foundation for subsequent tasks, such as cell nucleus segmentation.

**Figure 3:**
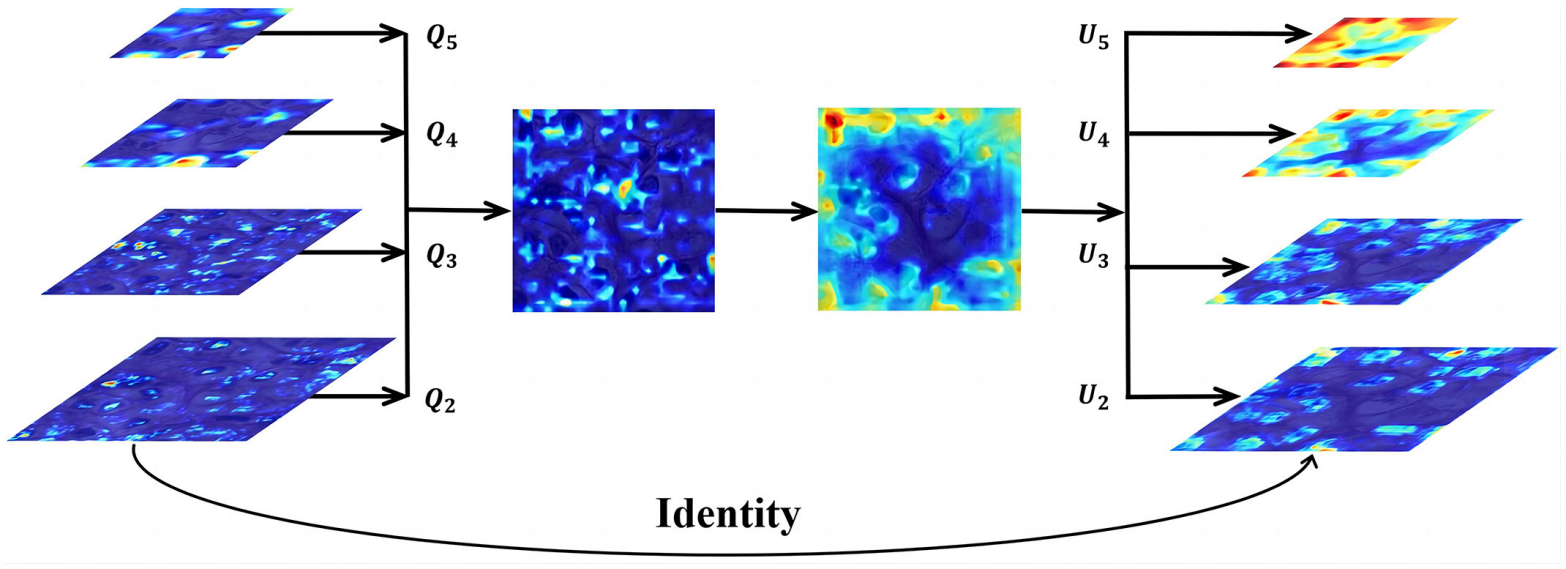
Feature integration module for feature level.*Q*_2_, *Q*_3_, *Q*_4_, and *Q*_5_ are multi-level features, and *U*_2_, *U*_3_, *U*_4_, and *U*_5_ are multi-level output signals.

Next, we utilize the balanced feature pyramid in our DMFNet to strengthen the multi-level features. The essential purpose of this module is to strengthen the multi-level features using the same deeply integrated balanced semantic features. It consists of four steps, namely rescaling, integrating, refining, and strengthening (Jiangmiao et al., 2019). The structure of this module is shown in Figure 3. To obtain balanced semantic features, we first resize the multi-level features {*Q*_2_, *Q*_3_, *Q*_4_, *Q*_5_} which have been generated by the FPN to the same size as *Q*_4_, and merge them to obtain *Q*_*int*_ with interpolation and max-pooling. The balanced semantic features can be expressed as:


(6) $$\begin{array}{l} Q_{int}=\frac{1}{L}\overset{l_{max}}{\underset{l_{min}}{\sum }}Q_{l} \end{array}$$

where *L* is the number of multi-level features; *Q*_*l*_ is the feature of resolution level *l*; and *l*_*min*_ and *l*_*max*_ are subtables representing the lowest and highest level indices, respectively. At this time, each resolution obtains equivalent information from the other resolutions.

Next, we use a non-local module (Xiaolong et al., 2018) to refine the balanced semantic features for more distinguishing features and better results. Non-local operations in deep neural networks are represented as:


(7) $$\begin{array}{l} U_{i}=\frac{1}{C\left(v\right)}\underset{\forall j}{\sum }f\left(v_{i},v_{j}\right)g\left(v_{j}\right) \end{array}$$

where *i* and *j* represent the indices of the output position and all possible associated positions, respectively; *v* indicates the input signal; U indicates the output signal, with the same size as *v*; and f (*v*_*i*_, *v*_*j*_) calculates the scalar between *i* and *j*. For example, the farther the distance between the positions of *i* and *j*, the smaller the value of the pairwise function *f*, which means that the position of *j* has less influence on *i*. *g*(*v*_*j*_) calculates the representation of the input signal at position *j* and *C*(*v*) is the normalization parameter.

Finally, the refined features of the four levels are added to the original features through interpolation or pooling to enhance the original features. We effectively create the semantic features of different layers using the BFP. This offers a better accuracy for nucleus segmentation.

### 2.3 Mask generation

**Figure 4:**
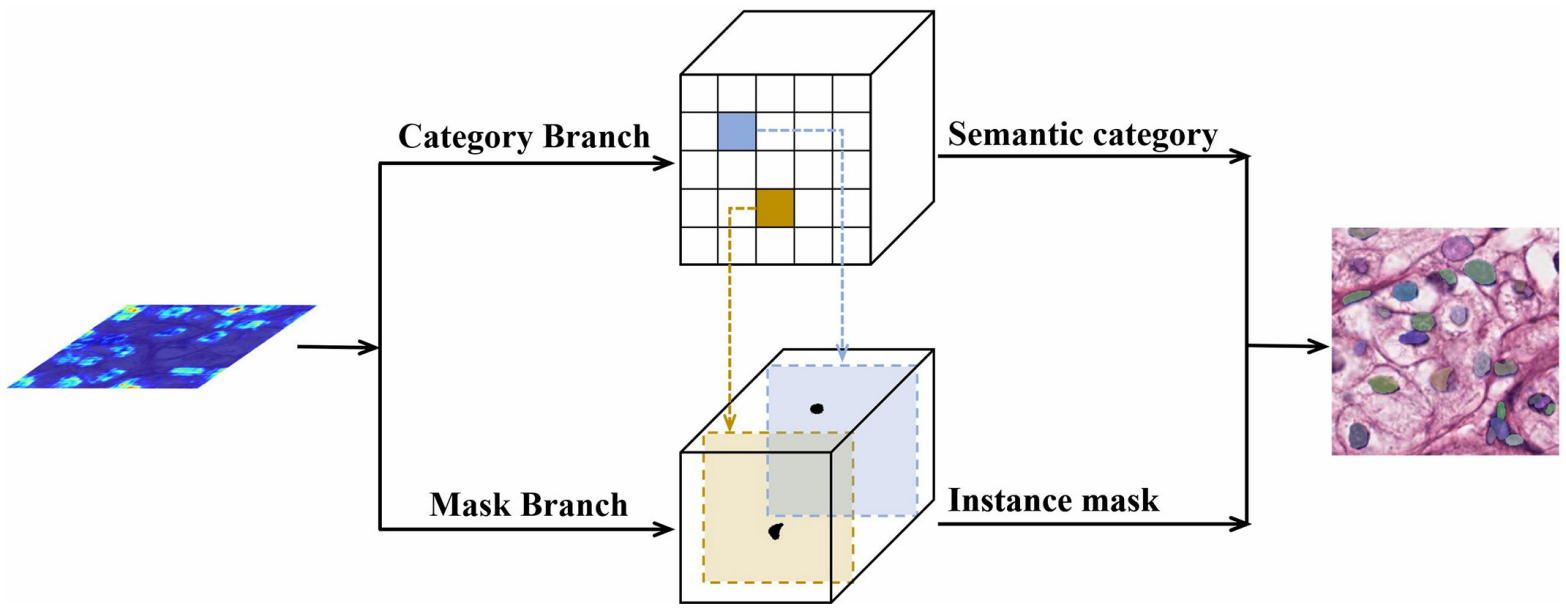
Mask generation network for mask level.

At the mask level, we further process the category prediction and instance mask generation. The pipeline for the same is shown in Figure 4.

We divide the picture into an *S*×*S* grid. The network output is divided into two branches, namely classification and mask branches. Simultaneous with the category prediction, each grid generates a corresponding instance mask. The size of the classification branch is *S*×*S*×*C*, where *C* is the number of categories. The mask branch size is *H*×*W*×*S*^2^, where *S*^2^ is the maximum number of instances predicted. When the center of the target object falls in the grid, the corresponding position of the classification branch and corresponding channel of the mask branch are responsible for the prediction of the object. For example, if the instance is allocated to the grid (*i, j*), then the channel *k* = *i*·*S*+*j* on the mask branch is responsible for predicting the mask of the target; each grid belongs to a single instance only. Finally, we use the non-maximum-suppression (NMS) algorithm to obtain the final results. Compared with a two-stage method, our one-stage method is simpler and can connect nucleus segmentation to a location to achieve better results.

The loss function includes two parts: category branch and mask branch. The loss function is as follows:


(8) $$\begin{array}{l} L_{DMF}=L_{\text{focal}}+\gamma L_{\text{m}} \end{array}$$

The Sigmoid activation function output is used here. *L*_*focal*_ represents the category branch, and uses the traditional semantic segmentation loss function Focal Loss (Shaoqing et al., 2017) to measure the gap between the predicted category and the ground truth. *L*_*m*_ is the loss function of the mask branch, specifically expressed as:


(9) $$\begin{array}{l} L_{\text{m}}=\frac{1}{N}\underset{k}{\sum }\beta p_{\left\{i,j>0\right\}}L_{\text{dice}} \end{array}$$

## 3 Results

### 3.1 Dataset

**Figure 5:**
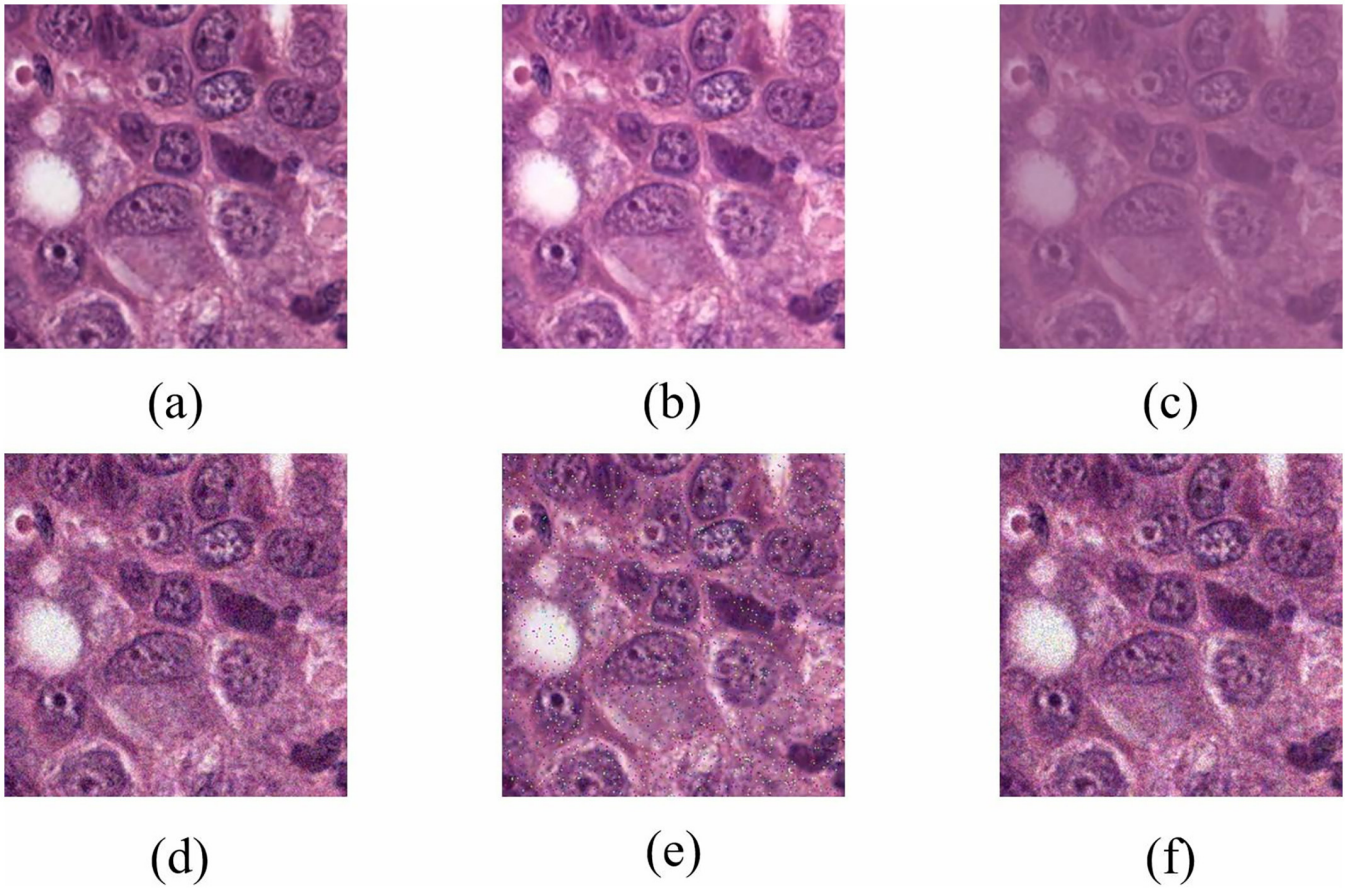
Representations of some image transformation schemes used. **(A)** Original. **(B)** Brightness enhancement. **(C)** Contrast reduction. **(D)** Gaussian noise. **(E)** Impulse noise. **(F)** Poisson noise.

MonuSeg stands for Multi-organ Nucleus Segmentation, and the dataset was published at the official satellite event of MICCAI 2018. The MoNuSeg 2018 dataset contains 30 tissue images and 21,623 annotated nuclear boundaries, each image of size 1, 000 × 1, 000 pixels (Kumar et al., 2017). The dataset used H&E-stained tissue slides digitized at 40x magnification and contained nuclei of varying sizes from seven different organs. These organs include the bladder, liver, breast, kidney, colon, prostate, and stomach. We cropped each image into 16 patches, and the size of each patch was 250 × 250 pixels. Specifically, we generated 480 images, including 352 training, 32 validation, and 96 test images. Furthermore, we used data augmentation to augment the size of the datasets and reduce overfitting. Before the image is input into the model, it will go through a channel composed of different data enhancement methods. Each enhancement method is set with a certain probability value and different enhancement factors. In other words, each image will follow the data in the channel. Augmentation is randomly combined with a set probability. Numerous image transformation schemes that were used include brightness enhancement, contrast reduction, Gaussian noise, impulse noise, and Poisson noise. Some representative examples of data augmentation are shown in Figure 5.

### 3.2 Implementation details

The DMFNet was implemented in PyTorch and trained on an NVIDIA Tesla V100 GPU with 32 GB of video memory. During the training, the mini-batch strategy was used to iteratively train the DMFNet for 200 epochs, and each iteration used two samples as a batch, with a total of 35,200 iterations. The validation set was evaluated after each training epoch. The network used batch normalization (Sergey and Christian, 2015) for regularization every time the weight was updated, and the stochastic gradient descent was used to update the model parameters. The learning rate, weight decay, and momentum were set to 0.0025, 0.0001, and 0.9, respectively.

### 3.3 Evaluation metrics

In object detection, the intersection-over-union (IoU) metric, which is the ratio of the intersection to the union of the prediction bounding box generated by the network and the original ground truth bounding box, was used. The evaluation method of instance segmentation was very similar to the evaluation method of object detection, with the difference being that the IoU of the mask was calculated in lieu of the IoU of the bounding box. In this study, precision and recall under a specific IoU threshold were considered as the evaluation indicators, and the expressions for the same are as follows:


(10) $$\begin{array}{l} Precision=\frac{TP}{TP+FP} \end{array}$$

(11) $$\begin{array}{l} Recall=\frac{TP}{TP+FN} \end{array}$$

where *TP* is the number of nuclei that are actually nuclei and are predicted by the model to be nuclei; *FP* is the number of nuclei that are actually background, but are predicted by the model; and *FN* is the number of nuclei that are actually nuclei, but not recognized as nuclei by the network. The *Precision* measures the proportion of samples that the model predicts as positive class (in nucleus segmentation, that is, predicted as nuclei) and are actually positive class among the samples predicted as positive class by the model. The *Recall* represents the proportion of samples that are actually positive class and are predicted as positive class by the model among the total number of actual positive class samples. The threshold of the IoU was calculated every 0.05 from 0.5 to 0.95 and the average precision (AP) was calculated every 0.05. The mean average precision (*mAP*) of all the results was used as the main indicator to report the results of the DMFNet. The *AP* under a specific IoU threshold was calculated as follows:


(12) $$\begin{array}{l} AP_{IoU=k}=\frac{1}{101}\underset{r\in R}{\sum }p_{\text{interp}}\left(r\right)=\frac{1}{101}\underset{r\in R}{\sum }\underset{\tilde{r}\cdot \tilde{r}\geq r}{\max}p\left(\tilde{r}\right) \end{array}$$

where *k* indicates the threshold in *K*:[0.5, 0.55, …, 0.90, 0.95], *r* denotes the recall, and *R*:[0, 0.01, 0.02, …, 0.99, 1.0], with an interval of 0.01 and a total of 101 values. $p\left(\tilde{r}\right)$ denotes the precision related to the recall rate $\tilde{r}$. To calculate the *AP* value at the ten thresholds, we considered the average value at the ten thresholds as the mean average precision (*mAP*). In addition, we also used the average recall (*AR*) as an evaluation metric, which was obtained by testing the mean *AR*_*IoU*_ = *k* of more than 10 IoU thresholds, and a maximum of the top 100 predicted masks were given. Similarly, we also considered the average value at the ten thresholds as the mean average recall (*mAR*). In this study, the task of the model was to identify only one category; therefore, *AR*_*IoU*_ = *k* at a specific threshold was equal to *R* in Equation 12. *mAP* is an important indicator for evaluating model performance, which measures the model's ability to accurately identify cell nuclei. A high *mAP* value indicates that the model performs well in accuracy and completeness. *AR* reflects the ability of the model to detect actual cell nuclei. In cell nucleus segmentation, high *AR* ensures that cell nucleus information is not missed, which can help detect diseased cells in early cancer screening in a timely manner. *mAR* measures the average performance of the model at different recall thresholds, calculating the average proportion of correctly predicted positive samples at multiple levels to the actual total number of positive samples. In addition, for *mAP* and *mAR*, we also used the following metric:

1. *AP*_50_: *AP* value over a single threshold of IoU = 0.50.
2. *AP*_75_: *AP* value over a single threshold of IoU = 0.75.
3. *AR*_50_: *AR* value over a single threshold of IoU = 0.50.
4. *AR*_75_: *AR* value over a single threshold of IoU = 0.75.

### 3.4 Ablation study

To verify the effectiveness of the feature extraction and feature integration modules, we used a network with and without those modules, embedded the same into the DMFNet, and then trained and evaluated them separately. To make an unbiased comparison, both these models used the same experiment and hyperparameter configuration. Table 1 summarizes the ablation studies on the effects of each module of the DMFNet.

**Table 1:** Effects of each module of the DMFNet (%).

***DCN***	***BFP***	***mAP***	***AP*_50_**	***AP*_75_**	***mAR***	***AR*_50_**	***AR*_75_**
		29.7	64.6	24.8	38.6	72.3	38.4
✓		34.9↑_5.2_	70.1↑_5.5_	32.5↑_7.7_	43.0↑_4.4_	77.3↑_5.0_	46.6↑_8.2_
	✓	31.6↑_1.9_	67.7↑_3.1_	26.1↑_1.3_	39.5↑_0.9_	73.9↑_1.6_	40.0↑_1.6_
✓	✓	37.8↑_8.1_	77.8↑_13.2_	33.6↑_8.8_	47.4↑_8.8_	85.3↑_13.0_	49.0↑_10.6_

As shown in Table 1, the DCN module was embedded in the SOLO, and the *AP* and *AR* scores of the segmented network were significantly improved, which means that the deformable feature extraction could better improve the transformation modeling capability of the model. This also proves that the deformable convolution had a higher accuracy for the irregular circle of the nucleus. When the BFP module was added, the *mAP* and *mAR* increased by 1.9% and 0.9%, respectively. This verifies that the multi-level feature integration played a key role. Specifically, the enhancement effect of the two modules was inconsistent; however, using the two modules at the same time yielded a better result, and it was proved that the DMFNet integrated the two modules effectively.

**Figure 6:**
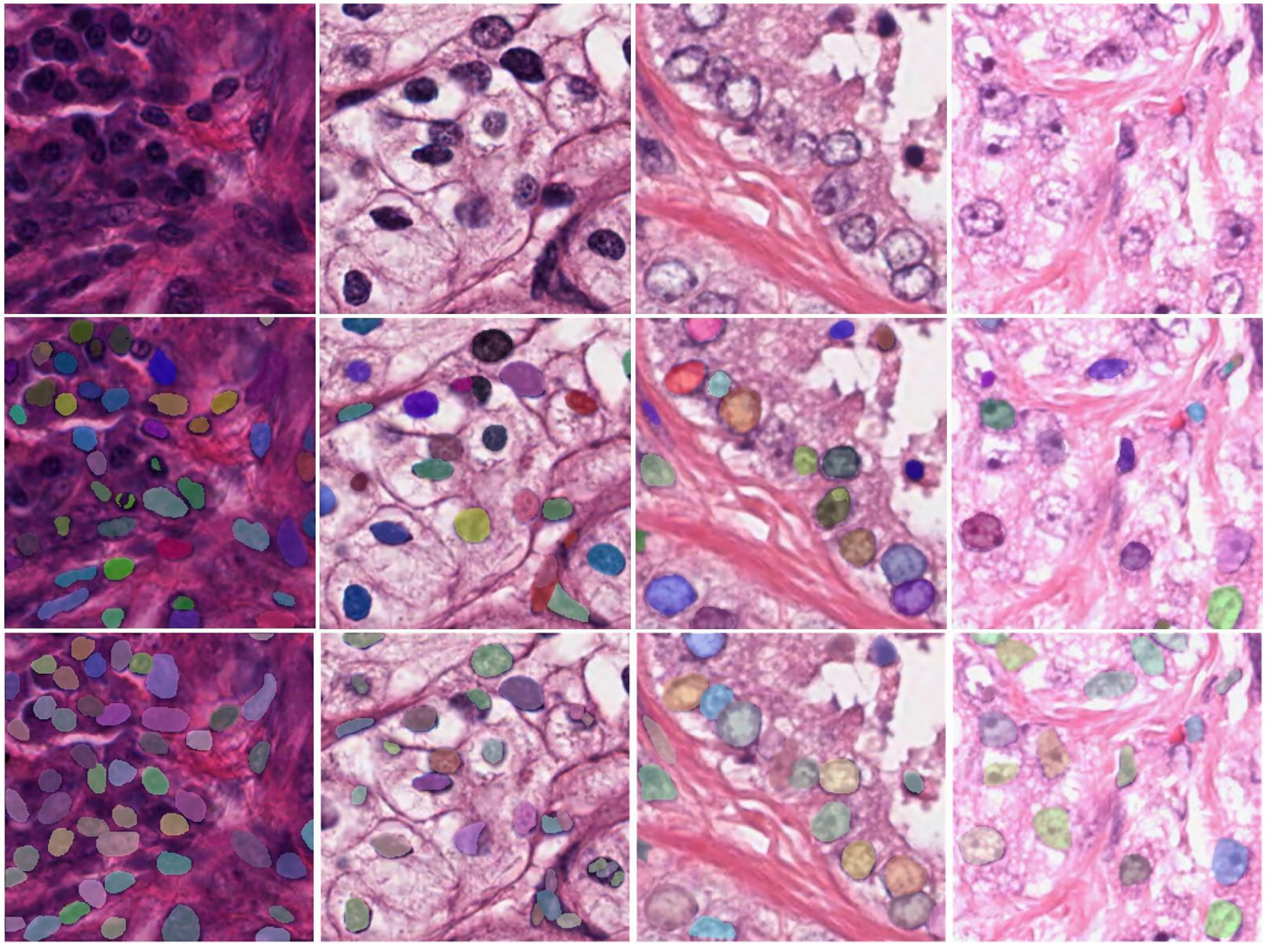
Visualization comparison between the DMFNets (with or without the two feature processing modules) and examples of segmentations model; the first row contains the original tissue images; the second row contains the images from the DMFNet without the two modules; while the third row contains the images from our proposed DMFNet.

Next, we visualized the ablation study and analyzed the details of our model. More typical examples are provided in Figure 6, including different stained nuclei and their masks. A model without the modules would result in errors, such as a small number of segmented nuclei and a lack of distinction between adjacent nuclei. The reason for this behavior was that the network did not completely learn the overall features and, hence, did not fully identify the edges of the features. In contrast, the DMFNet could segment more nuclei, correctly segment adjacent nuclei, and also segment each nucleus more completely. This means that our model offers advantages in nuclei edge extraction and global feature integration, and it is also proved that the two modules played a significant role.

### 3.5 Comparisons

To verify the effectiveness of the DMFNet on nuclei segmentation, we compared the proposed model with a few state-of-the-art methods, with the same experimental configuration. Table 2 lists the instance segmentation results of each of these methods. Compared to the existing baseline methods, for example, Mask R-CNN (Kaiming et al., 2017), TensorMask (Xinlei et al., 2019), and PolarMask (Xie et al., 2020), the proposed method yielded better results than the state-of-the-art methods. The DMFNet achieved the best performance on the MoNuSeg 2018 dataset, with its *mAP* and *mAR* approaching 37.8% and 47.4%, respectively. Furthermore, when the IoU threshold was 0.50 and 0.75, our method yielded a better performance, and both *AP* and *AR* were the best.

**Table 2:** Effects of each module of the DMFNet (%).

**Method**	***mAP***	***AP_50_***	***AP_75_***	***mAR***	***AR_50_***	***AR_75_***
PolarMask	21.7	52.4	14.5	31.8	64.1	29.4
Mask R-CNN	30.3	67.8	24.1	40.9	77.4	40.2
TensorMask	31.4	67.4	27.1	39.8	74.0	39.7
SOLO	29.7	64.6	24.8	38.6	72.3	38.4
DMFNet	37.8	77.8	33.6	47.4	85.3	49.0

**Figure 7:**
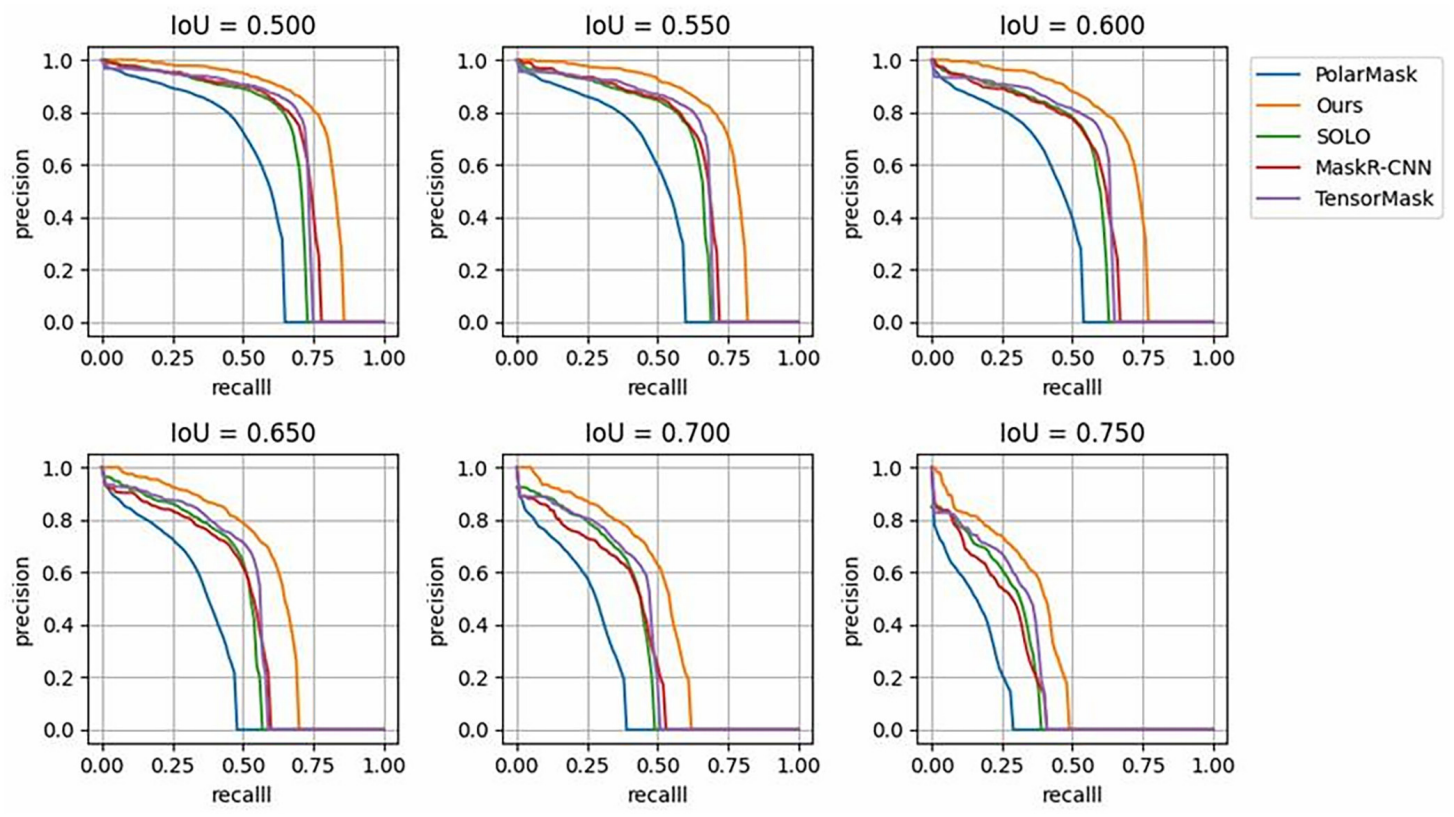
P-R Curves of state-of-the-art models over different IoU thresholds, compared with those of the proposed DMFNet.

Figure 7 shows the segmentation P-R curves (smoothed) of five models with 10 IoU thresholds ranging from 0.5 to 0.75 with an interval of 0.05. Figure 7 shows that with a change in the IoU threshold, the trend of the model was approximately the same, and the corresponding P-R curve gradually approached the coordinate axis; however, the closing speed of the P-R curve of the proposed model was slower than that of the other models. This proves that the prediction result of the proposed DMFNet had a higher score and better overall quality.

## 4 Discussion

Changes in nuclear morphology are closely related to the growth state of tumors. For nearly 150 years, changes in nuclear morphology have been the gold standard for cancer diagnosis, so it is critical to understand single nuclear instances. With the development of digital pathology, the emergence of electronic pathology pictures helps pathologists get rid of microscopes, but it is very cumbersome and difficult to browse and observe pathological nuclei on the computer. The main method for pathologists to quantify nuclei is subjective estimation, which cannot be done precise quantification.

In digital pathology research, instantiating nuclei can help pathologists understand the structure of individual nuclei. Quantitative analysis of the spatial distribution of nuclear clusters and the number of mitoses, which are key factors in cancer diagnosis and prognosis. In practice, there are independent nuclei and clusters of nuclei that are clustered together. Although the semantic segmentation of nuclei has an excellent effect and can extract nuclei from pathological images, it is not suitable for independent study of nuclei clusters or adjacent nuclei. Scene of the nucleus in a cell.

For the above reasons, we propose a model DMFNet to segment pathological image nuclei from the perspective of instance segmentation. DMFNet analyzes the process of nucleus instance segmentation at both feature and segmentation levels. At the feature level, we added a feature extraction module for the diverse morphological characteristics of nuclei, which enhanced the model's transformation and modeling capabilities to better sample target instances. In nucleus segmentation, both detail features and semantic features play a very important role, so we propose to use the feature integration module to integrate and enhance the two features. At the mask level, we replace the traditional methods using anchor boxes or clustering with a one-stage location-based instance segmentation method, making the model simpler. Experiments show that the method in this paper can effectively improve the accuracy of nucleus instance segmentation.

In summary, both feature extraction and feature integration modules can improve the accuracy of nucleus segmentation; moreover, their combination can further improve the performance, which not only validates that their combination is suitable for the task of nucleus segmentation, but also shows that they differ from feature level Aspects enhance the segmentation task. Therefore, we can use these two modules simultaneously in the application scenario of nucleus segmentation to achieve optimal segmentation results.

In the field of medical image processing, the development of automatic annotation technologies is of critical significance for improving the efficiency of clinical applications. The DMFNet model in this study demonstrates remarkable potential in the automatic annotation of medical images. By achieving precise segmentation of cell nuclei, the model can automatically identify and annotate key information such as the location and boundaries of the nuclei. This greatly reduces the workload associated with manual annotation. Traditional manual annotation methods require significant time and effort from pathologists, especially when handling large-scale medical image data. In contrast, the DMFNet model can rapidly and accurately perform the annotation task. For example, annotating pathological slide images containing numerous cell nuclei may take several hours or even days manually, while the DMFNet model can complete the preliminary annotation in a much shorter time, achieving high accuracy. This not only improves annotation efficiency but also provides timely and reliable data support for subsequent clinical diagnosis and research, thereby promising to enhance overall clinical application efficiency and provide robust support for early disease diagnosis and precision treatment.

In terms of practical clinical applications, this nuclear cell segmentation model holds great potential. In the process of cancer diagnosis, accurate nuclear cell segmentation is a crucial step. Currently, the incidence rate of cancer remains at a relatively high level, and early diagnosis is of great significance for improving patients' survival rates and quality of life. By enhancing the accuracy of nuclear cell instance segmentation, this model can provide pathologists with more accurate and detailed information about nuclear cells. For example, in the diagnosis of common cancers such as breast cancer and lung cancer, pathologists can utilize this model to observe the morphology, size, and distribution of nuclear cells more clearly, thereby making a more accurate judgment on the benign or malignant nature of the tumor. This is of great importance for the early detection of tiny tumors, the determination of cancer staging, and the formulation of personalized treatment plans. Moreover, during the follow-up after cancer treatment, this model can also be used to monitor changes in the morphology of nuclear cells, enabling the timely detection of signs of cancer recurrence.

We analyzed the problem of nuclei segmentation in pathological images and proposed a model for nuclei segmentation, but with the change of datasets and the development of medical images, the model still needs to be further optimized. Although the nucleus segmentation dataset we used is a well received work in recent years, in the process of analyzing the edges, it is found that the labeling of the edges is not fine enough and the amount of training data is relatively small. Therefore, transfer learning should be performed in combination with newly released datasets to improve the robustness and accuracy of the model. We only segment the nuclei in the pathological images without considering the types of nuclei. In tumor tissues, the cells included not only cancer cells, but also stromal cells, lymphocytes, macrophages, etc. Recent studies have shown that tumor cell nuclei Stromal cell interactions are involved in tumor progression and metastasis. In the following work, the fine-grained classification of the research cells is also needed, and the cells are divided into tumor cells, stromal cells, lymphocytes, etc.

## 5 Conclusions

In this study, we proposed an innovative model, DMFNet, which holds significant value in clinical applications. This model is mainly used for nuclear segmentation from digital pathology images of different organs, a function that is of great significance for the clinical diagnosis and treatment of diseases such as cancer. In clinical practice, the accuracy of nuclear segmentation is crucial for determining the nature and development stage of tumors. We conducted a detailed analysis of the nuclear segmentation process and made targeted improvements to key components such as feature extraction, fusion, and template generation. Through these enhancements, DMFNet effectively combines DCN, BFP, and SOLO, significantly improving the performance of the segmentation network.

However, the DMFNet model still has certain limitations. At the methodological level, although it exhibits good performance on the existing dataset, with the development of medical imaging technology and the emergence of new datasets, the model may face adaptability issues. From the perspective of network structure, although the performance has been improved through module combination, when dealing with large-scale, high-resolution pathological images, the problem of excessive consumption of computational resources is rather prominent, which affects the running efficiency of the model to a certain extent. In terms of feature extraction, the expression of features such as the morphology and texture of the cell nucleus is not rich enough, making it difficult to capture some subtle but critical pathological features, which may thus affect the accuracy of segmentation. In the process of feature fusion, the fusion method of different hierarchical features is not optimal, resulting in information loss or redundancy, leading to the underutilization of some features. The template generation process is not flexible enough in adapting to the diverse morphological distributions of cell nuclei, and the processing ability of the model is limited when facing complex pathological situations.

In the future, we will further improve the model from the directions of enhancing model performance (such as optimizing feature extraction, fusion, and template generation), expanding application scenarios (to more organ diseases and integrating with clinical processes), and increasing model interpretability (visualization and constructing explanatory models). When the DMFNet was applied to the MoNuSeg 2018 dataset, the experimental results clearly demonstrated the performance advantages of this method over some existing methods. This implies that in actual clinical scenarios, pathologists can utilize this model to more accurately extract nuclear information from pathological images. Meanwhile, during the follow-up of diseases, this model also helps in continuously monitoring the changes of cell nuclei, enabling the timely detection of disease progression or recurrence.

In summary, the DMFNet model provides important support for clinical diagnosis and treatment and has remarkable clinical value.

## Data Availability

The original contributions presented in the study are included in the article/supplementary material, further inquiries can be directed to the corresponding author.
